# Racial/Ethnic Differences and Effects of Clinical/Socioeconomic Factors on Time from Diagnosis to Treatment in Pancreatic Cancer

**DOI:** 10.1007/s12029-025-01188-x

**Published:** 2025-02-15

**Authors:** Anush Sridharan, Efrat Dotan, Marianna Dorta, Navya Vemula, Elizabeth Handorf, Mengying Deng, Ashley Renning, Kristen Sorice, Lauren Laderman, Kate Whittington, Edna Cukierman, Igor Astsaturov, Namrata Vijayvergia, Joshua E. Meyer, Sanjay S. Reddy, Shannon M. Lynch

**Affiliations:** 1 Lewis Katz School of Medicine, Temple University, Philadelphia, PA USA; 2 Population Studies Facility, Fox Chase Cancer Center, Philadelphia, PA USA; 3 Fox Chase Cancer Center, Cancer Prevention and Control, Young Pavilion P4159, 333 Cottman Avenue, Philadelphia, PA 19111 USA; 4 Program of Cancer Signaling and Epigenetic, Fox Chase Cancer Center, Philadelphia, PA USA; 5 Department of Hematology/Oncology, Fox Chase Cancer Center, Philadelphia, PA USA; 6 Department of Medical Oncology, Fox Chase Cancer Center, Philadelphia, PA USA; 7 Department of Radiation Oncology, Fox Chase Cancer Center, Philadelphia, PA USA; 8 Department of Surgical Oncology, Fox Chase Cancer Center, Philadelphia, PA USA

**Keywords:** Pancreatic cancer, Survival, Social determinants, Neighborhood, Health disparities

## Abstract

**Purpose:**

Five-year survival for pancreatic adenocarcinoma (PDAC) is < 10% but can vary by a patient’s race, socioeconomic status (SES), and the factors related to the neighborhood where a patient lives (nSES) . Prolonged time from diagnosis to first treatment (T2T) is another important disparity indicator. Here, we examined the effect of race, nSES, and patient-level clinical factors on T2T and survival in metastatic PDAC (mPDAC) patients.

**Methods:**

Patients with mPDAC treated at an academic cancer center between 2010 and 2018 (*n* = 334) were evaluated for nSES measures related to racial concentration, neighborhood deprivation, stability, immigration status, and transportation access from the US Census. We assessed and reported the effects of nSES and patient-level variables (age, race, gender, Charlson Comorbidity Index (CCI), etc.) on T2T and survival using univariate and multivariate Cox proportional hazards regression, hazard ratios (HR), confidence intervals (CI).

**Results:**

82.9% of the patients were White; 17.1% were Black. Median T2T was 26 days with no significant difference in T2T and survival by race. In multivariable models, no nSES variables were significantly associated with T2T. T2T did not significantly impact survival; however, receipt of chemotherapy (HR = 0.14 [95% CI = 0.06, 0.30]) was associated with better survival outcomes.

**Conclusion:**

Among patients with mPDAC, T2T was not associated with race/ethnic disparities or survival in a mostly White, high SES population treated at a comprehensive cancer center. Future investigations into pancreatic cancer disparities may be warranted in other hospital settings and in larger, more diverse study samples.

## Introduction

Pancreatic cancer (PDAC) is the fifth leading cause of cancer death in the USA [1]. Between 2006 and 2015, the incidence of PDAC increased by 1% per year [2]. For all stages of PDAC, the relative 5-year survival rate is 9%, with surgery as the only chance for cure [2, 3]. However, less than 20% of PDAC patients can undergo surgery, as the disease usually is metastatic at diagnosis [2]. It has been predicted that by 2030, pancreatic cancer will be the second leading cause of cancer death in the United States [1, 2].

Longer time to treatment (T2T) is often associated with worse survival [4–6]. A study found that time from diagnosis to treatment of greater than 29 days resulted in worse PDAC survival outcomes [7]. However, this and a majority of studies to date have included or focused on earlier stage PDAC populations, when realistically most patients are initially diagnosed with metastatic disease. Further, racial/ethnic differences in T2T have not been explored in the metastatic setting [7]. Health disparities, particularly varying rates of disease progression and differences in treatment by race/ethnicity, are observed in PDAC populations. Black patients have significantly higher incidence and mortality rates compared to non-Hispanic White and Hispanic/Latinx patients [8]. Black patients are also less likely to receive surgical treatment for early stage PDAC compared to White patients [9, 10]. Further, T2T has been found to be longer in Black patients when compared to White patients across all stages of PDAC [11].

One potential factor that could be influencing differences in outcomes of patients with PDAC is socioeconomic status (SES). SES is often defined as an individual’s education, income, employment, and insurance status [12]. Patients with higher SES are more likely to receive treatment [13] and have shorter time to treatment [14] based on studies in other cancer types; however, this has not been well-explored among PDAC patients. In some studies, when individual SES measures are not available, neighborhood socioeconomic status (nSES) measures have been used as surrogate measures for a patient’s SES [15]. To note, studies have also shown that nSES exerts independent effects on cancer outcomes and pancreatic cancer survival more specifically [16]. However, very few studies have comprehensively evaluated the impact of nSES on time from diagnosis to first treatment and the impact of T2T on metastatic pancreatic cancer outcomes in the context of race/ethnicity and other important clinical and social factors [4, 12, 17, 18].

We conducted a retrospective study to evaluate racial/ethnic differences in T2T and survival among patients with metastatic PDAC (mPDAC) treated at a comprehensive cancer center between 2010 and 2018. We evaluated differences by race/ethnicity for the effects of patient demographic and clinical factors as well as neighborhood-level factors on T2T and overall survival (OS).

## Methods

### Study Population

Patient data was obtained retrospectively from medical records of mPDAC patients at tertiary academic cancer center after review and approval by the Institutional Review Board. Patients diagnosed with mPDAC between 2010 and 2018 were retrospectively identified using the institution’s tumor registry, based on ICD-9 coding. Cases with incomplete medical record data and/or missing address information were excluded. The total study population consisted of 334 patients and only included White and Black patients due to the small quantity of patients from other racial and ethnic origins. Residential addresses of mPDAC patients were geocoded up to the census tract level and assigned a Federal Information Processing Standard (FIPS) geocode [19, 20] using Arc GIS software v. 10.6. (ESRI; Redlands, CA). Patient information was merged to nSES variables (see below) via the FIPS code using Stata v. 11.0 (College Station, TX). Patients living in the same census tract were assumed to have the same neighborhood characteristics. There were 298 unique census tracts included in this analysis.

### Outcome

The primary clinical outcomes of interest were T2T and OS, both calculated using the date of mPDAC diagnosis as the time origin. The first treatment was identified based on clinician-reported treatment dates. Information regarding date of death was obtained through the tumor registry. Patients who were living as of December 31, 2019, or the date of the last follow-up were considered censored. Differences in time to treatment and survival were compared by race/ethnicity.

### Patient Variables

To ensure data accuracy, patient charts were abstracted and quality controlled by two independent reviewers. Based on associations with PDAC risk or survival in prior literature, the following patient characteristics and clinical factors were included in the analysis: age, sex, race (Black, White), body mass index (BMI), diabetes (yes/no), tobacco use (yes/no), self-identified Jewish ancestry, family history of PDAC, marital status, number of chemotherapy agents received, and type of first treatment (no treatment, palliative radiation, chemotherapy, and chemoradiation) [10, 21–25]. Age-adjusted Charlson Comorbidity Index (CCI) was calculated for each patient by utilizing the published scoring method [19]. The majority of patients in this dataset had insurance coverage, which was not included in the analysis.

### nSES Variables

nSES variables were derived from the US Census American Community Survey (ACS) at the census tract level between 2011 and 2015 [25]. nSES variables were linked to patient-level medical records by converting address information into a geocode at the census tract level. nSES variables that have been found to be associated with outcomes related to cancer and other health conditions [4, 13, 17, 18, 26] were selected for inclusion in this study, including deprivation, racial concentration transportation access (the proportion of residents owning a vehicle), immigration (the proportion of foreign-born residents), and neighborhood stability (the proportion residents living in the same house as one year prior) [16].

Deprivation was measured using a previously validated composite SES measure (Yost Index), created by principal component analysis of seven indicator variables. The index is categorized into quintiles based on the census tract values for the overall state with 1 being the highest level of deprivation and 5 being the lowest level of deprivation. Racial concentration (RC) was defined using the Index of Concentration at the Extremes (ICE-index). ICE-race measures the degree of isolation/separation of racial/ethnic groups in a neighborhood, with a standard score ranging from − 1 (concentration of Non-Hispanic Blacks (NHB)) to 1 (concentration of Non-Hispanic Whites (NHW)). This measure was categorized into quantiles, based on prior literature [26]. Transportation access, immigration, and stability were analyzed as continuous variables.

### Statistical Analysis

We compared treatment, clinical/demographic factors, nSES variables, and T2T across race using standard summary statistics including percentages, means, and medians. Median T2T and OS were calculated via Kaplan–Meier curves with log-rank tests (Tables 1 and 2). The effect of patient-level variables and nSES variables on T2T was assessed in multivariable models (Table 4). All models were estimated using Cox proportional hazards (PH) regression models, with robust standard errors to account for correlation within census tracts [27, 28]. Similarly, multivariable Cox PH regressions were also used to evaluate mPDAC survival in patients with additional adjustments made for the first treatment type and time to treat (Table 4). To reduce potential biases, we excluded patients who did not receive any type of treatment from the OS analysis. All multivariable models were adjusted using race as a covariate. Significant variables known to impact pancreatic cancer in prior literature were included in these multivariable models. Hazard ratios and 95% confidence intervals (95% CI) were analyzed. Variables with *p*-values < 0.05 were considered significant.

**Table 1:** Patient characteristics at diagnosis of metastatic disease

Characteristics	Overall (*n* = 334)	White (*n* = 277)	Black (*n* = 57)	*p*-value^1^
**Time from diagnosis of metastatic disease to treatment (in days)**				
Mean (SD)	29 (19)	28 (18)	32 (22)	
Median [95% CI]	26 [24–28]	26 [23–28]	27 [23–31]	0.18
**Time from diagnosis of metastatic disease to survival (in months)**				
Mean (SD)	12.00 (13.38)	11.80 (12.83)	12.99 (15.96)	
Median [95% CI]	9.40 [8.21–10.3]	9.40 [8.21–10.5]	9.72 [7.43–12.0]	0.49
**Age (in years)**				**0.008**
Mean (SD)	65.56 (10.24)	66.23 (10.36)	62.30 (9.05)	
Median [range]	66.90 [27.53–90.75]	67.33 [27.53–90.75]	63.09 [45.05–80.83]	
**Sex, *N* (%)**				**0.007**
Male	189 (56.6%)	166 (59.9%)	23 (40.4%)	
Female	145 (43.4%)	111 (40.1%)	34 (59.6%)	
**Marital status, *N* (%)**				0.21
Not married	120 (35.9%)	88 (31.8%)	32 (56.1%)	
Married	214 (64.1%)	189 (68.2%)	25 (43.9%)	
**BMI**				0.51
Mean (SD)	27.08 (5.52)	26.99 (5.22)	27.51 (6.86)	
Median [range]	26.60 [16.01–66.24]	26.34 [16.01–50.87]	27.33 [17.70–66.24]	
**Patient vital status, *N* (%)**				
Alive	13 (3.9%)	9 (3.2%)	4 (7.0%)	0.18
Deceased	321 (96.1%)	268 (96.8%)	53 (93.0%)	
**Number of chemo agents received**				0.89
Mean (SD)	3.42 (1.47)	3.42 (1.50)	3.40 (1.37)	
Median [range]	3 [1–6]	3 [1–6]	3 [1–6]	
Missing (*n*)	30	23	7	
**Type of first treatment, *N* (%)**				0.83
No treatment	28 (8.4%)	22 (7.9%)	6 (10.5%)	
Palliative radiation	83 (24.9%)	68 (24.5%)	15 (26.3%)	
Chemotherapy	218 (65.3%)	182 (65.7%)	36 (63.2%)	
Chemoradiation	5 (1.5%)	5 (1.8%)	0 (0.0%)	
**History of tobacco use, *N* (%)**				0.62
No	120 (35.9%)	97 (35.0%)	23 (40.4%)	
Yes	214 (64.1%)	180 (65.0%)	34 (59.6%)	
**Charlson Comorbidity Index (CCI)**				0.59
Mean (SD)	8.91 (1.53)	8.89 (1.43)	9.01 (1.93)	
Median [range]	9.00 [6.00–17.00]	9.00 [6.00–14.00]	9.00 [6.00–17.00]	
**Family history of pancreatic cancer, *N* (%)**				0.45
No	286 (85.6%)	239 (86.3%)	47 (82.5%)	
Yes	48 (14.4%)	38 (13.7%)	10 (17.5%)	
**History of diabetes**				0.36
No	228 (68.3%)	192 (69.3%)	36 (63.2%)	
Yes	106 (31.7%)	85 (30.7%)	21 (36.8%)	
**Initial metastatic site(s)**				
Liver	269 (80.5%)	218 (78.7%)	51 (89.5%)	**0.06**
Lung	68 (20.4%)	55 (19.9%)	13 (22.8%)	0.61
Peritoneal	77 (23.1%)	70 (25.3%)	7 (12.3%)	0.03
Bone	0 (0.0%)	0 (0.0%)	0 (0.0%)	-
Local	6 (1.8%)	5 (1.8%)	1 (1.8%)	0.97
Other	24 (7.2%)	21 (7.6%)	3 (5.3%)	0.53
**Jewish ancestry**				0.12
No	313 (93.7%)	257 (92.8%)	56 (98.2%)	
Yes	21 (6.3%)	20 (7.2%)	1 (1.8%)	

Bolded values indicate a statistically significant difference between the race cohorts (*p* < 0.05)

**Table 2:** Neighborhood socioeconomic status (nSES) variables at diagnosis of metastatic disease

Variables	Overall (*n* = 334)	White (*n* = 277)	Black (*n* = 57)	*p*-value^1^
**Racial concentration**				**0.001**
Q1-concentration of NHW	40 (12.0%)	40 (14.4%)	0 (0.0%)	
Q2	53 (15.9%)	51 (18.4%)	2 (3.5%)	
Q3	94 (28.1%)	89 (32.1%)	5 (8.8%)	
Q4	70 (21.0%)	66 (23.8%)	4 (7.0%)	
Q5-concentration of NHB	77 (23.1%)	31 (11.2%)	46 (80.7%)	
**Neighborhood deprivation (Yost Index)**				** < 0.001**
Q1-low SES	31 (9.3%)	13 (4.7%)	18 (31.6%)	
Q2	54 (16.2%)	41 (14.8%)	13 (22.8%)	
Q3	54 (16.2%)	43 (15.5%)	11 (19.3%)	
Q4	73 (21.9%)	68 (24.5%)	5 (8.8%)	
Q5-High SES	122 (36.5%)	112 (40.4%)	10 (17.5%)	
**Stability (percent living in the same house as 1 year ago)**				**0.01**
Mean (SD)	90.29 (5.32)	90.62 (4.87)	88.68 (6.94)	
Median [range]	91.01 [55.43–99.34]	91.37 [67.48–99.07]	89.67 [55.43–99.34]	
**Immigration (proportion of foreign-born residents)**				0.99
Mean (SD)	10.60 (8.58)	10.60 (8.62)	10.60 (8.49)	
Median [range]	8.61 [0.00–42.11]	8.68 [0.00–42.11]	7.55 [0.84–32.82]	
**Transportation access (percent owning a vehicle)**				** < 0.001**
Mean (SD)	89.80 (11.46)	92.66 (6.59)	75.86 (18.12)	
Median [range]	93.94 [35.86–100.00]	94.73 [63.42–100.00]	79.33 [35.86–100.00]	

*Q1-Q5*, quartiles/quantiles 1–5; *SES*, socioeconomic status; *NHW*, Non-Hispanic White; *NHB*, Non-Hispanic Black

Bolded values indicate a statistically significant difference between the race cohorts (*p* < 0.05)

## Results

The study population consisted of 334 patients with mPDAC, of which 56.6% were males and 82.9% were White. Black patients were more likely to be female (59.6%). The median age of the total study population was 67 years old (range, 28–91 years); the median age of Black patients was 63 years old. Overall, 96.1% of participants were deceased by the end of the study. About 64% of all patients had a history of tobacco use, 14.4% had a family history of PDAC, 35.9% were not married, and 6.3% were of Jewish descent. In regard to the first treatment, about 88% received chemotherapy, 1.5% received chemoradiation, 2.4% received palliative radiation, and 8.4% received no treatment. 10.5% of Black patients received no treatment compared to 7.9% of White patients (*p* = 0.28). Furthermore, 5.3% of Black patients received palliative radiation compared to 1.8% of White patients (*p* = 0.28). The median number of chemotherapy agents received during the course of treatment was three (3) agents, and this was the same across race groups. The number of agents ranged from 1 to 11, although most patients ranged between 1 and 6 agents; there was one patient in this cohort who received up to 11 agents, which contributed to this wider overall range. In addition, there was a significant difference in initial metastatic sites with a greater incidence of peritoneal metastasis in White patients (25.3%) compared to Black patients (12.3%) (*p* = 0.03). The Charlson Comorbidity Index (median = 9.0 [6.00–17.00]) and BMI (median = 26.6 [16.01–66.24]) were similar across racial groups. The median time from diagnosis of mPDAC to treatment was 26 days (range, 0–120 days), and the median time from treatment to death or last follow-up was 9.29 months (range, 0.13–121.26 months) and similar across racial groups (Table 1). There was no difference in time from diagnosis to first treatment among Black and White (median = 26 days (range, 0–120 days) patients. There was also no difference in OS between White (median = 9.33 months; range, 0.13–121.26) and Black patients (median = 8.71 months; range, 0.32–91.89) (*p* = 0.52).

Comparing nSES variables on this study’s mPDAC patient population, the majority of patients (~ 58%) lived in neighborhoods of higher SES scores (low deprivation, Q4 and Q5), with White patients comprising about 92% of this group (*p* ≤ 0.001). Meanwhile, within the Black patient population, about 54% of patients lived in low SES neighborhoods (high deprivation, Q1 and Q2) (*p* ≤ 0.001). With regard to racial concentration, 80.7% of Black patients were more likely to live in neighborhoods with a high concentration of Non-Hispanic Black (NHB) residents, with only 3.5% living in high concentration of Non-Hispanic White (NHW) neighborhoods. Neighborhood stability and transportation access were lower among Black patients compared to White patients (*p* = 0.01 and *p* ≤ 0.001, respectively), and immigration measures were relatively similar across race groups (*p* = 0.99).

In univariate analysis (Table 3), the following variables were associated with longer T2T: higher age at time of mPDAC diagnosis (HR = 0.99 [95% CI = 0.98, 0.99]; *p* = 0.02), higher CCI (HR = 0.92 [95% CI = 0.85, 0.99]; *p* = 0.02), and ECOG performance status of 1 (HR = 0.98 [95% CI = 0.75, 1.28]; *p* < 0.001). Meanwhile, Jewish ancestry (HR = 1.40 [95% CI = 1.03, 1.91]; *p* = 0.03) and transportation access (the proportion of residents owning a vehicle) (HR = 1.01 [95% CI = 1.00, 1.02]; *p* = 0.02) were associated with shorter T2T. However, no variables were significantly associated with T2T in multivariable analysis with adjustment for both patient and neighborhood variables (Tables 3 and 4); there was some attenuation of effects (slight improvements in HRs) after adjustment in the multivariable model. Further, the model fit, or AIC, was similar for T2T models adjusted only for patient-level variables (AIC = 3006.91—where Jewish ancestry remained significant; Tables 3 and 4) compared to patient plus nSES models (AIC = 3008.4; Tables 3 and 4).

**Table 3:** Association of patient-level and neighborhood socioeconomic status (nSES) variables with time to first treatment and pancreatic cancer survival in univariate models

Variables	Time to treatment—patient and nSES variable model			Pancreatic cancer survival—patient and nSES variables model		
	HR	95% CI	*p*-value	HR	95% CI	*p*-value
**Age**	0.99	[0.97, 0.99]	**0.02**	1.02	[1.00, 1.03]	** < 0.01**
**Sex**						
Female	Ref			Ref		
Male	1.19	[0.94, 1.50]	0.14	0.94	[0.74, 1.18]	0.62
**Race**						
White	Ref			Ref		
Black	0.79	[0.94, 1.50]	0.14	0.89	[0.63, 1.26]	0.53
**Marital status**						
Married	Ref			Ref		
Not married	1.24	[0.97, 1.58]	0.08	0.80	[0.63, 0.99]	**0.05**
**Family history of pancreatic cancer**						
No	Ref			Ref		
Yes	0.95	[0.68, 1.33]	0.77	0.88	[0.63, 1.22]	0.44
**Jewish ancestry**						
No	Ref			Ref		
Yes	1.40	[1.03, 1.91]	**0.03**	1.44	[1.01, 2.04]	**0.04**
**Charlson Comorbidity Index (CCI)**	0.92	[0.85, 0.99]	**0.02**	1.09	[1.02, 1.17]	**0.01**
**ECOG performance status**						
1	0.98	[0.75, 1.28]	** < 0.01**			
2	0.56	[0.34, 0.94]	NA			
3	0.11	[0.03, 0.49]	NA			
4	0.00	[0.00, 0.00]	NA			
99	1.09	[0.67, 1.79]	NA			
**Time to treat**				1.05	[0.83, 1.33]	0.68
**Chemotherapy**						
No				Ref		
Yes				0.14	[0.07, 0.30]	** < 0.01**
**Neighborhood variables**						
Deprivation index*						
Q5	Ref			Ref		
Q1	0.77	[0.56, 1.07]	0.12	1.00	[0.64, 1.59]	0.75
Q2	0.69	[0.48, 0.99]	0.17	0.94	[0.68, 1.37]	0.75
Q3	0.97	[0.69, 1.38]	0.89	1.09	[0.82, 1.46]	0.54
Q4	0.85	[0.62, 1.18]	0.36	0.87	[0.63, 1.20]	0.39
**Immigration**	0.98	[0.97, 1.00]	0.09	1.00	[0.98, 1.01]	0.99
**Transportation access**	1.01	[1.00, 1.02]	**0.02**	1.00	[0.99, 1.01]	0.76

*HR* hazard ratio, *CI* confidence interval

^*^Q1, quartile 1 (most deprived), Q2, Q3, Q4, quartile 4/quintile 5 (least deprived); *Bolded values indicate a statistically significant difference between the race cohorts (*p* < 0.05)

**Table 4:** Association of patient-level and neighborhood socioeconomic status (nSES) variables with time to first treatment and pancreatic cancer survival in multivariable models

Variables	Time to treatment—patient and nSES variable model			Pancreatic cancer survival—patient and nSES variables model		
	HR	95% CI	*p*-value	HR	95% CI	*p*-value
**Age**	0.99	[0.97, 1.01]	0.25	1.01	[1.00, 1.03]	**0.03**
**Sex**						
Female	Ref			Ref		
Male	1.12	[0.88, 1.42]	0.34	0.91	[0.72, 1.15]	0.43
**Race**						
White	Ref			Ref		
Black	0.92	[0.59, 1.40]	0.69	0.92	[0.61, 1.40]	0.71
**Marital status**						
Married	Ref			Ref		
Not married	1.13	[0.89, 1.44]	0.31	0.80	[0.63, 1.02]	0.07
**Family history of pancreatic cancer**						
No	Ref			Ref		
Yes	1.03	[0.74, 1.46]	0.84	0.71	[0.49, 1.05]	0.08
**Jewish ancestry**						
No	Ref			Ref		
Yes	1.38	[0.99, 1.92]	0.05	1.50	[0.99, 2.27]	0.05
**Charlson Comorbidity Index (CCI)**	0.94	[0.83, 1.06]	0.35	1.04	[0.92, 1.17]	0.56
**Time to treat**				1.04	[0.82, 1.32]	0.70
**Chemotherapy**						
No				Ref		
Yes				0.14	[0.06, 0.30]	** < 0.01**
**Neighborhood variables**						
Deprivation index*						
Q5	Ref			Ref		
Q1	0.91	[0.56, 1.47]	0.74	1.41	[0.72, 2.77]	0.31
Q2	0.75	[0.50, 1.14]	0.17	1.02	[0.68, 1.55]	0.91
Q3	1.03	[0.77, 1.47]	0.84	1.25	[0.91, 1.71]	0.17
Q4	0.87	[0.63, 1.18]	0.41	0.92	[0.67, 1.26]	0.62
**Immigration**	0.98	[0.97, 1.00]	0.12	0.99	[0.97, 1.01]	0.47
**Transportation access**	1.00	[0.99, 1.02]	0.37	1.00	[0.98, 1.02]	0.39

*HR* hazard ratio, *CI* confidence interval

^*^Q1, quartile 1 (most deprived), Q2, Q3, Q4, quartile 4/quintile 5 (least deprived); *Bolded values indicate a statistically significant difference between the race cohorts (*p* < 0.05)

In univariate analyses for OS (Table 3), higher age at time of mPDAC diagnosis (HR = 1.01 [95% CI = 1.00, 1.03]; *p* = 0.0008), having Jewish ancestry (HR = 1.43 [95% CI = 1.01, 2.03]; *p* = 0.04), and higher CCI (HR = 1.09 [95% CI = 1.01, 1.16]; *p* = 0.01) were associated with shorter OS. Time to treatment was not associated with OS (HR = 1.05 [95% CI = 0.83, 1.33]; *p* = 0.68). Longer OS was seen in patients who received any course of chemotherapy treatment versus those who did not (HR = 0.14 [95% CI = 0.07, 0.29]; *p* < 0.001), increasing total number of chemotherapy agents received (HR = 0.73 [95% CI = 0.67, 0.80]; *p* < 0.001), and being married (HR = 0.80 [95% CI = 0.64, 0.99]; *p* = 0.05). In multivariate analysis for OS, increasing age was associated with shorter OS (HR = 1.02 [95% CI = 1.00, 1.03]; *p* = 0.03), and receiving any course of chemotherapy treatment was associated with longer OS (HR = 0.14 [95% CI = 0.06, 0.03]; *p* < 0.01) (Tables 3 and 4). The model fit, or AIC, was slightly better for mPDAC survival models including patient-only factors (AIC = 2803.15) compared to models with patient-level and nSES variables (AIC = 2811.17).

## Discussion

This study sought to comprehensively evaluate disparities in mPDAC clinical outcomes by race/ethnicity. We evaluated the length of time from mPDAC diagnosis to first treatment (T2T) and overall mPDAC survival (OS) in a cohort of patients treated at a comprehensive cancer center using both clinical and nSES as indicators. Black compared to White patients were more likely to live in low SES (high deprivation) neighborhoods, but nSES indicators were not associated with T2T. In fact, no significant difference in T2T and OS was found between Black and White patients in univariate and multivariable analysis. In univariate analyses, age at mPDAC diagnosis, Jewish ancestry, CCI, deprivation, and transportation access were found to be related to T2T; however, these associations did not persist in multivariable models.

Our results demonstrate limited disparities among our patient population. The higher percentage of the study population with some type of insurance coverage and a middle to higher nSES background makes it difficult to stratify or assess differences by nSES or to explore the intersection between race/ethnicity and SES*.* The majority of the patients in this study came from the Philadelphia area, where despite living in lower SES circumstances, residents have physical access to quality tertiary care with high-volume centers in close proximity. Studies have shown that high-volume centers often have better treatment outcomes and long-term survival for pancreatic cancer compared to low-volume centers [26, 29], though it should be noted we are investigating a single center in this study. Further, all the patients included in these analyses had some form of health insurance (private, Medicare, or Medicaid), which is a critical factor affecting access to care, timely treatment initiation, and survival. Thus, taken together, these factors appear to explain the lack of disparity in T2T in this cohort of patients with appropriate access to quality cancer care. Additional studies in larger, multiethnic cohorts from rural, urban, and both low and high-volume cancer centers are still needed, particularly because T2T could serve as a potential evaluation metric to ensure patients are receiving equal access to care.

Time to first treatment also was not associated with overall survival in univariate and multivariable models. The median T2T was 26 days, which is less than a prior investigation finding that time to first treatment in excess of 29 days is associated with worse pancreatic cancer outcomes [7]. This could explain why time to the first treatment did not impact survival in this study. The only factors impacting survival after multivariable adjustment included higher age at mPDAC diagnosis and receiving any course of chemotherapy treatment. It is not surprising that younger age and receiving treatment with chemotherapy improves survival, similar to prior studies [11].

Neighborhood SES variables were not significantly associated with survival in models with T2T and did not improve multivariable model fit; however, after adjustment for nSES in OS multivariable models, it did appear to attenuate and help explain the observed univariate effects related to transportation access, CCI, and the overall survival outcome. This could be because the majority of our patients come from mid to higher level SES backgrounds. T2T was not significant in OS multivariable models but also had an attenuating effect on variables significant in univariate analysis. CCI was not associated with survival in multivariable models. Prior studies have found that treatment at academic centers can increase time from initial diagnosis to treatment due to the complexity of patients that are seen at academic centers [30]. Thus, it is possible that accounting for multiple variables, for instance, CCI and time to treatment, in the same model resulted in the lack of effect of CCI on survival.

Our study had a number of limitations to report. While there were 277 White participants, there were only 57 Black participants; thus, findings by race/ethnicity should be interpreted with caution. Given the small sample size, we were unable to run multivariable models stratified by race. We also did not observe significant differences in the number of chemotherapy agents received or in the type of first treatment by race/ethnicity, suggesting that similar care was received, regardless of race. Time to treatment is a disparity-related indicator often associated with access to care, where longer time to treatment has been associated with lack of health coverage [3]. Further, insurance coverage has been found to influence the types of therapies chosen and the readiness with which patients approach treatment after receiving a diagnosis [11]. The higher percentage of the study population with some type of insurance coverage and a middle to higher nSES background makes it difficult to stratify or assess differences by nSES. To begin to address disparities identified in this analysis, additional grant funding related to increasing diversity and representation in clinical research studies at our institute has been procured. It is possible that racial/ethnic differences were not observed in metastatic patients for time to first treatment and survival because, at baseline, patients seen at our center generally have insurance coverage, live in higher resource neighborhoods, and have access to quality health care [31]. A recently published study by Madnick and colleagues also found that even in the context of a patient’s race/ethnicity, clinical factors, and SES measures, neighborhood stability was significantly associated with pancreatic cancer survival [32, 33]. Thus, our findings are likely not generalizable to the general public or other practice settings.

## Conclusion

Overall, taking into account prior literature related to racial/ethnic differences in pancreatic cancer time to first treatment and survival, this study suggests that once patients access clinical care at a tertiary, comprehensive cancer center, that disparities in treatment may be reduced. This suggests an opportunity to reduce disparities if we increase access opportunities for disadvantaged patients. Future investigations in larger, more ethnically diverse samples are needed to understand mPDAC disparities and to evaluate whether improvements in access to tertiary care can significantly reduce these disparities.

## Data Availability

Data cannot be shared for the purpose of maintaining ethical boundaries and protecting the privacy of the patients involved in this research. There is summary-level data available within this manuscript.
